# Finding Closure: A Closer Look at the Gestalt Law of Closure in Convolutional Neural Networks

**DOI:** 10.1007/s42113-025-00251-x

**Published:** 2025-06-16

**Authors:** Yuyan Zhang, Derya Soydaner, Lisa Koßmann, Fatemeh Behrad, Johan Wagemans

**Affiliations:** 1 Department of Computer Science, University of Leuven (KU Leuven), Leuven, 3000 Belgium; 2 Leiden Institute of Advanced Computer Science, Leiden University, Leiden, 2333 CC The Netherlands; 3 Department of Brain & Cognition, University of Leuven (KU Leuven), Leuven, 3000 Belgium; 4 Leuven.AI, KU Leuven Institute for AI, Leuven, 3000 Belgium

**Keywords:** Neural networks, Gestalt laws, Closure, Deep learning

## Abstract

The human brain has an inherent ability to fill in gaps to perceive figures as complete wholes, even when parts are missing or fragmented. This phenomenon, known as Closure in psychology, is one of the Gestalt laws of perceptual organization. Given the role of Closure in human perception, we investigate whether neural networks exhibit similar functional behavior in object recognition. While the neural substrates of Gestalt principles are thought to involve feedback mechanisms in the brain, convolutional neural networks (CNNs) rely on feedforward architectures. Despite this, we focus on the functional comparison—specifically, object recognition—rather than the underlying mechanisms. We investigate whether CNNs can parallel the human ability to perform Closure. Exploring this crucial visual skill in neural networks can highlight their (dis)similarity to human vision. Recent studies have examined the Closure effect in neural networks, but typically focus on a limited selection of CNNs and yield divergent findings. To address these gaps, we present a systematic framework to investigate Closure. We introduce well-curated datasets designed to test for Closure effects, including both modal and amodal completion. We then conduct experiments on nine CNNs employing different measurements. Our comprehensive analysis reveals that VGG16 and DenseNet-121 exhibit the Closure effect, while other CNNs show variable results. This finding is significant for fields such as AI, Neuroscience, and Psychology, as it bridges understanding across disciplines. By blending insights from psychology and neural network research, we offer a unique perspective that enhances transparency in neural networks.

## Introduction

Tasks that come naturally to humans are still challenging for AI models, despite the increasing integration of AI-based technologies across various domains. The ease with which neural networks can be confused, illustrated by examples such as texture bias an image of a cat textured as an elephant (Geirhos et al., 2018), serves as an amusing yet stark reminder of their limitations compared to the human brain’s capabilities. While we can easily recognize faces, even a slight perturbation in an image can deceive a neural network (Szegedy et al., 2013; Goodfellow et al., 2015), illustrating the significant gap that still exists in mimicking human visual perception.

**Fig. 1:**
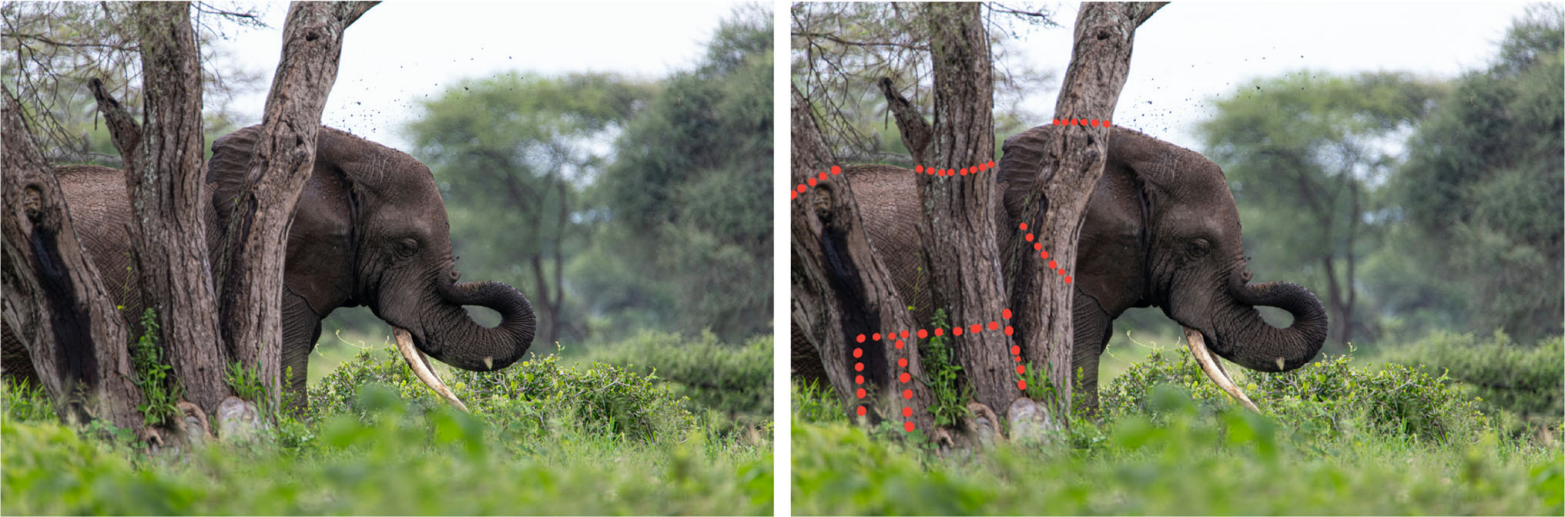
Two photos show an elephant behind trees. While the visible front part alone provides sufficient cues for humans to recognize the elephant, the Principle of Closure helps complete the elephant’s contour behind the trees, illustrated by the superimposed red dots on the right. However, current CNNs struggle to perform Closure, making it occasionally challenging for them to classify the elephant. We investigate whether CNNs can parallel the human ability to perform Closure. Image credit: Glen Michaelsen, via Unsplash

A major milestone in the study of visual perception was the development of the principles of perceptual grouping, commonly known as the “Gestalt Laws” (Wertheimer, 1938; Palmer, 2002; Wagemans et al., 2012a; Wagemans, 2018). These principles explain how observers tend to perceive certain elements as grouped together, while others are perceived as separate. The classic grouping principles include proximity, similarity, common fate, good continuation, symmetry, parallelism, and closure. Among these, Closure stands out as a principle stating that individuals perceive incomplete shapes as complete, forming a coherent whole. This is due to contour completion, which can happen when an object is perceived to be behind an occluder (amodal completion) or when stimulus characteristics give rise to an illusory contour (modal completion). Figure 1 shows a real-world example of amodal completion. Closure is an integral part of how the human visual system makes sense of the oftentimes ambiguous input of our visual reality. It particularly aids in figure-ground segregation; the process which entails visually separating a figure from the background. For this, the contour and especially its shape are important factors (Peterson & Salvagio, 2008). The phenomenology and theory of what is often referred to as “The Closure Effect,” has been demonstrated and replicated by many vision scientists (Wagemans et al., 2012a, b).

Recent studies on CNNs exploring this phenomenon show varying results: some indicate that they exhibit Closure within certain thresholds and limitations (Amanatiadis et al., 2018; Ehrensperger et al., 2019; Kim et al., 2021), while others do not Baker et al. (2018); Zhang et al. (2024). This lack of consensus and the diverse findings have prompted our research to further investigate and settle the debate on whether CNNs exhibit this Gestalt principle.

Our study focuses on CNNs for two main reasons. First, it allows us to address the gaps identified in previous research conducted on CNNs, particularly the mixed findings regarding their ability to perform Closure. Second, CNNs operate differently from other neural networks, such as Vision Transformers (ViTs) (Dosovitskiy et al., 2020), which incorporate attention mechanisms. Given the complex interrelationships between attention mechanisms and perceptual grouping in the human brain (Wu et al., 2023), ViTs require a separate investigation. By clarifying the role of closure in CNNs, we provide the necessary foundation for future research.

Although humans and CNNs both perform object recognition, humans naturally exhibit Closure. This study begins by examining whether current CNNs also exhibit this perceptual skill. Since CNNs aim to perform tasks similar to those of humans, our hypothesis is that they should develop abilities comparable to human visual perception, particularly at the fundamental level. Modeling CNNs after the human visual system could therefore enhance their performance in computer vision. Although CNNs operate differently from the human brain—which relies on recurrent interactions in cortical layers—understanding whether CNNs can approximate this perceptual task, despite their distinct architectures, could bridge the gap between human cognitive processes and machine learning techniques. Our main contributions are as follows^1^:

- We present well-curated datasets designed to test Closure effect, with carefully controlled conditions to evaluate both modal and amodal completion in neural networks.
- We design psychology-based experiments to assess whether CNNs exhibit the Closure effect, addressing limitations and resolving the lack of consensus in the literature.
- We conduct a detailed analysis of various CNNs, expanding the range of models examined in previous studies.
- Our research sheds light on the Gestalt law of Closure in CNNs, contributing to our understanding of their ability to mimic human visual perception.

## Related Work

An early study (Amanatiadis et al., 2018) investigated six core Gestalt laws, including closure, by training AlexNet (Krizhevsky et al., 2017) and Inception V1 (GoogLeNet) (Szegedy et al., 2015) on the MNIST (Lecun et al., 1998) and ImageNet (Deng et al., 2009) datasets. Closure was assessed using occlusion percentage, with findings showing that the Closure principle is effective in CNNs up to approximately 30% occlusion, beyond which the models’ performance decreases. While this study provides valuable insights, it is limited by its focus on older architectures. The lack of experimentation with modern CNNs raises questions about the generalizability of their findings. Additionally, relying solely on occlusion percentage as a metric overlooks key distinctions between modal and amodal completion.

Building on this accuracy-based method, a recent study (Zhang et al., 2024) expanded the investigation by training a wider range of CNNs on complete polygons and testing their performance on incomplete ones. The findings suggest that CNNs do not exhibit the Closure effect under the specific measurement and dataset used. However, further investigation is needed to draw conclusions. Despite incorporating modern architectures, this study remains limited by its reliance on a single metric—occlusion percentage—and does not address distinctions between modal and amodal completion.

Another study fine-tuned AlexNet to classify wireframes and Kanizsa squares as “fat” and “thin” in a shape discrimination task (Baker et al., 2018). They concluded that neural networks do not perceive illusory contours. While this study provides an interesting exploration of shape discrimination using wireframes and Kanizsa squares, its broad conclusion—that neural networks do not perceive illusory contours—is based on a single model and a narrowly focused task. This limited scope raises concerns about the generalizability of their claim.

Subsequent studies, such as Biscione and Bowers (2023), employed a similarity-based method and examined a broader range of models, including ResNet-152 (He et al., 2016) and DenseNet-201 (Huang et al., 2017). They reported mixed evidence of perceptual grouping. This study broadens the scope by including more CNNs in its experiments. However, its focus on the overarching concept of perceptual grouping, rather than a specific Gestalt law like Closure, may explain the “mixed” findings. A more detailed and targeted analysis would be necessary to draw clearer conclusions.

Another study analyzed the classification performance of AlexNet and Inception V1 on Kanizsa triangles (we discuss Kanizsa triangles in the next section) and modified triangles with removed edge sections, reporting evidence of Closure (Ehrensperger et al., 2019). This study, while reporting evidence of Closure, relies solely on AlexNet and Inception V1 evaluated on a classification task.

Further research examined Inception and a simple CNN, both trained on natural images and found evidence of Closure on synthetic edge fragments (Kim et al., 2021). Testing these CNNs on incomplete triangles revealed that they were more likely to be recognized as similar to complete triangles rather than disordered fragments. However, their focus on Inception and a simple CNN limits its generalizability. Additionally, their dataset requires significant modifications, as discussed later in our experiments, which may impact the validity of their findings.

While not directly aligned with our research focus, a noteworthy study tested a latent noise segmentation model on various datasets reflecting Gestalt laws, including Closure (Lonnqvist et al., 2023). Similarly, another recent study introduced a toolbox for testing deep neural networks against 30 psychological findings (Biscione et al., 2024). However, their toolbox includes only amodal completion based on Rensink and Enns (1998), which features a square alongside a disk and does not test deep neural networks on this dataset.

Notably, a model’s ability to identify partially occluded objects is not a definitive proof that they are doing so by applying the principle of Closure. Instead of model and or amodal completion, successful classification could be accomplished through reliance on local features or texture cues. Successful classification despite occlusion is, however, a valid first assessment, as failure would rule out the presence of Closure in the model.

## Methodology

### Problem Definition

**Fig. 2:**
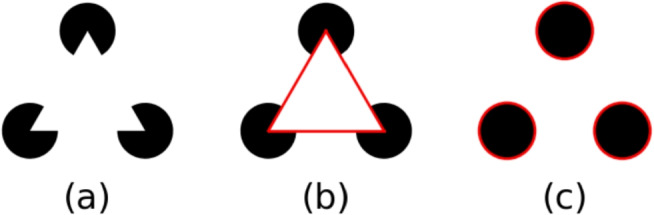
(**a**) An example of a Kanizsa triangle. The image consists of three black fragments with a white triangle perceived in front them. (**b**) Modal completion and (**c**) amodal completion in the Kanizsa triangle

**Fig. 3:**
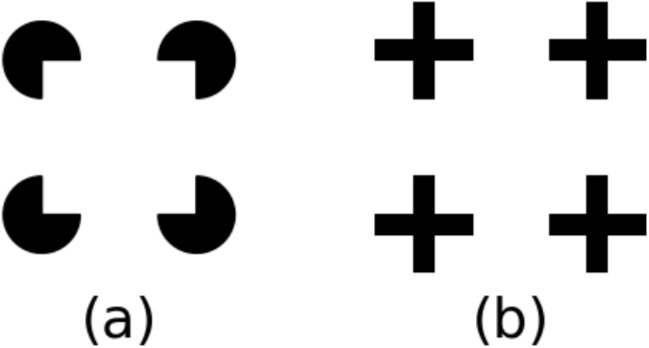
Illustration of modal completion. In both (**a**) and (**b**), the edges are similarly aligned, yet an illusory square emerges only in (**a**) due to modal completion. In contrast, (**b**) does not induce the perception of a shape, highlighting that edge alignment alone is not sufficient to facilitate modal completion (Kogo et al., 2010)

The Gestalt principle of Closure, the automatic tendency for the human visual system to organize disjointed elements into a whole, is an integral part of human visual perception (Wagemans et al., 2012a). According to the Closure principle, the visual system completes contours to create closed shapes, through processes referred to as contour integration (distinct elements are integrated into a contour forming a shape) or contour completion (integrating smooth contours that are separated due to occlusion or camouflage) (Wagemans et al., 2012a). There are two forms of contour completion: amodal and modal completion (Wagemans et al., 2006). During amodal completion, the object is completed behind an occluder. This has often been studied using line drawings and observers report the strong intuition that the object continues behind the occluder (Wagemans et al., 2006). Modal completion includes so-called illusory contours and surfaces, which are triggered by specific stimulus characteristics. A prominent example of modal completion is the Kanizsa Triangle (Kanizsa, 1955) shown in Fig. 2. Here, the specific properties of the “pac-man” shaped circles, particularly their missing parts, give rise to a white triangle superimposed on three black circles. Note, however, while distinctions between the two processes can be made, they often go together. In the Kanizsa triangle, for example, amodal completion is also present, in the form of the circles being completed behind the triangle (Wagemans et al., 2006). A similar interplay can be observed in the Kanizsa square, which further demonstrates the role of modal completion. Notably, modal completion is not simply the result of edge alignment. As illustrated in Fig. 3, the alignment of edges is the same in both (a) and (b), but the illusory square only emerges in (a), due to modal completion.

Line drawings and Kanizsa triangles may appear to be specific instances that bear little importance in the complex visual reality of everyday life. However, the processes revealed by studying them are highly important for understanding how the human visual system organizes the—sometimes ambiguous—input from the optic apparatus into objects (Wagemans et al., 2012a) and enables us to understand whole scenes in a few milliseconds (Potter et al., 2014). Therefore, investigating this essential human visual “skill” in neural networks, can not only shed light on their comparability with humans but its integration in the processing stream could also potentially increase their detection and classification abilities (Zhang et al., 2024).

### Convolutional Neural Networks

CNNs have revolutionized computer vision by learning feature hierarchies from low to high-level patterns. Pioneering models such as AlexNet (Krizhevsky et al., 2017) and VGG16 (Simonyan & Zisserman, 2015) have achieved high accuracy in image recognition tasks. However, their use of numerous small filters has resulted in high computational complexity. Later advances addressed this issue: ResNet (He et al., 2016), SqueezeNet (Iandola et al., 2016), and DenseNet (Huang et al., 2017) employ residual connections, efficient filters, and dense connectivity, respectively, to achieve comparable or superior accuracy while reducing computational costs. Other progress include Inception networks (Szegedy et al., 2015) and ShuffleNet (Zhang et al., 2018). Inception networks use filters of varying sizes within each layer to capture a wider range of spatial features. ShuffleNet enhances information flow through channel shuffling, significantly improving performance. Additionally, EfficientNet (Tan & Le, 2019) uses compound scaling to optimize network depth, width, and resolution based on resource constraints. MobileNet (Howard et al., 2017), featuring depthwise separable convolutions, is particularly well-suited for mobile devices due to its efficient processing.

It is important to clarify that by “computational complexity,” we refer to the number of parameters in a model. For example, ResNet18, SqueezeNet, and DenseNet have fewer parameters than AlexNet and VGG16, resulting in lower memory and computational requirements. While deeper variants exist (e.g., ResNet-152 has around 60 M parameters, comparable to AlexNet), our focus is on the main architectures rather than their variations to highlight their fundamental contributions to the field.

**Fig. 4:**
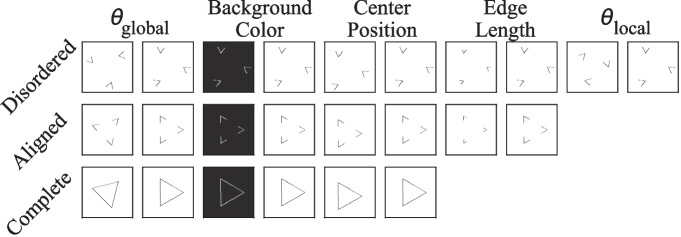
Examples of the three groups of images used in the first experiment, with parameters matching those specified by Kim et al. (2021)

While all these convolutional neural networks share the fundamental principle of extracting features from images using convolutional layers, their architectures exhibit subtle yet crucial differences. To comprehensively investigate Closure, our experiment deliberately incorporates a diverse range of network architectures, allowing us to observe how Closure manifests across different design choices. In our experiments, we utilized pre-trained CNNs, freezing all their layers. All networks in this study were pre-trained on the ImageNet (Deng et al., 2009) image set for an object classification task. We refrained from training them on completion tasks, as training on natural images resembles the development of visual perception in humans. Humans exhibit the Gestalt law of closure without explicit training. Our research explores whether these neural networks can similarly exhibit such inherent capabilities. We obtain our results based on the output of the last convolutional layer in VGG16, SqueezeNet V1.1, ShuffleNet V2, and MobileNet V3; the final MBCConv6 layer for EfficientNet B0; Mixed 7c layer for Inception V3; the last fully-connected layer for AlexNet; the final average pooling layer for ResNet-50; and the final dense block for DenseNet-121.

## Experiments

### Experiment 1: The Similarity-Based Method

Our first experiment builds upon the work of Kim et al. (2021), who demonstrated that CNNs trained for natural image classification do perform Closure. We begin by replicating their study, generating the same dataset with two necessary modifications and extending the methodology to a broader range of CNNs. This part focuses on Triangle Segment Completion, using displays of edge fragments. This extended replication enables us to determine whether Closure can be observed in a broader range of CNNs beyond just the Inception network and sets a baseline for our subsequent analysis. We then adapt the experiment to include Kanizsa triangles—a condition not previously explored in the referenced study.

#### Triangle Segment Completion

#### Dataset

The dataset used by Kim et al. (2021) contains 992 different images, which are categorized into three groups: complete triangles, aligned triangle fragments, and disordered triangle fragments. The triangles and fragments differ in global orientations ($\theta _{global}$), background colors, center positions, edge length (defined as the length of the visible edge of each fragment in the image; 3, 8, 13, 18, 24, or 29 pixels, respectively) and local orientations ($\theta _{local}$) (see Fig. 4). $\theta _{global}$ is the degree to which the triangle rotates around its center in the plane, with values from 0°, to 105°in steps of 15°. ($\theta _{local}$), describing the degree to which the triangle fragment rotates around the corresponding vertex, can be 72°, 144°, 216°, or 288°. All fragments in an image share the same $\theta _{local}$ and have a size of $150 \times 150$. The distance between any two vertices in any image is 116 pixels. Overall, there are 32 complete, 192 aligned, and 768 disordered triangles. We replicate their dataset with two notable modifications: (1) the size of each image is set to $300 \times 300$. (2) the possible positions of the center of a triangle were changed to (150, 150) and (134, 134). This is because in some conditions (e.g., when the edge length is 29 pixels, $\theta _{global}$ = 0°, and $\theta _{local}$ = 144°), a disordered triangle fragment would go over the border of the canvas and the resulting image would not be able to show the whole fragment.

#### Measurement

We adopt the same measurement for assessing the Closure effect as implemented by Kim et al. (2021) to ensure a fair comparison across different CNN models. This measurement is shown below, where *Similarity* represents cosine similarity:

1 $$\begin{aligned} \text {Closure Measure}= &  \text {Similarity}(\text {aligned}, \text {complete}) \nonumber \\ &  - \text {Similarity}(\text {disordered}, \text {complete}) \end{aligned}$$

2 $$\begin{aligned} Similarity(\textbf{x}, \textbf{y})= &  \frac{f(\textbf{x}) f(\textbf{y})^T}{|f(\textbf{x})| | f(\textbf{y})|} \end{aligned}$$

where $f(\textbf{x})$ is the output vector of a layer in the model.

If the model exhibits Closure, we would expect the similarity value between a pair of aligned and complete triangles to be greater than that between a pair of disordered and complete triangles, resulting in a relatively large difference.

#### Results and Discussion

**Fig. 5:**
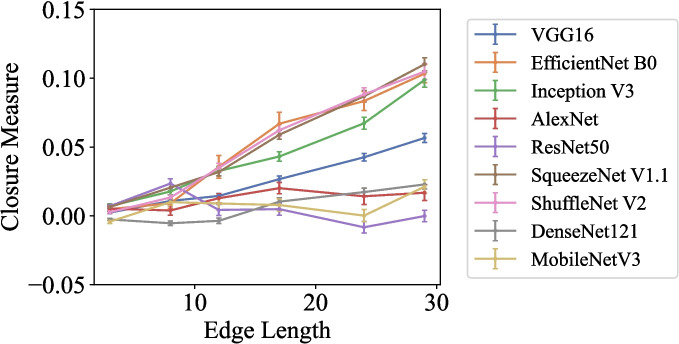
Closure measure plotted against edge length (in pixels) for Experiment 1, “Triangle Segment Completion.” The Closure measure ranges from −1 to 1, with larger values indicating a stronger Closure effect

The average values of the Closure measure for AlexNet, ResNet-50, DenseNet-121, and MobileNet V3 are around 0 for all edge lengths (Fig. 5). However, the Closure measure increases with edge length increases for VGG16, EfficientNet B0, Inception V3, SqueezeNet V1.1, and ShuffleNet V2. Most of their Closure values exceed 0 when the edge length is 13 pixels or more.

To further explore whether there is a significant increasing trend in the Closure measure, we conduct a general multivariate linear regression analysis for each model. The predicted value is the Closure measure, and the predictors are the edge length, the $\theta _{global}$, the background color, the centre position, and the $\theta _{local}$. Among these variables, the Closure measure and the edge length are treated as continuous, while all other variables are nominal. The results show that although all regression models are significant ($p <.001$), only those for VGG16, EfficientNet B0, Inception V3, SqueezeNet V1.1, and ShuffleNet V2 have at least moderate effect sizes (adjusted $R^2 >.40$). For these models, the edge length can significantly predict the Closure measure when other variables are controlled ($b >.0030$, $p <.001$). The regression models for AlexNet and DenseNet-121 have small effect sizes (adjusted $R^2 <.30$) and the predicting effect of the edge length is significant in the two regression models ($b =.0003$, $p <.001$ for AlexNet, and $b =.0006$, $p <.001$ for DenseNet-121).

To summarize, our first experiment not only replicates the results of Kim et al. (2021) on Inception V3 but also demonstrates the Closure effect in VGG16, EfficientNet B0, SqueezeNet V1.1, ShuffleNet V2, AlexNet, and DenseNet-121, although the effects of the last two models might be small. On the other hand, ResNet-50, and MobileNet V3 do not show the Closure effect. This difference in the results could be due to the different structures of the models. While the first layers, which extract local features, are similar across CNN architectures, the models primarily differ in how they extract global features. However, the type of Closure or the measurement method may also play a role. Further experiments exploring these possibilities are conducted in the following sections.

#### Kanizsa Triangles

#### Dataset

**Fig. 6:**
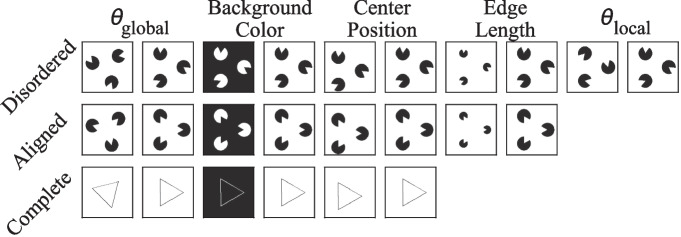
Examples of the three groups of images featuring Kanizsa triangles used in our experiment

The dataset for this experiment has the same size and settings as the previous one except that it includes complete triangles, valid Kanizsa triangles (aligned triangle fragments), and invalid Kanizsa triangles (disordered triangle fragments), as illustrated in Fig. 6. While all other parameters remain consistent with the previous experiment, the valid Kanizsa triangles in this dataset are composed of three incomplete disks, rather than triangle fragments.

**Fig. 7:**
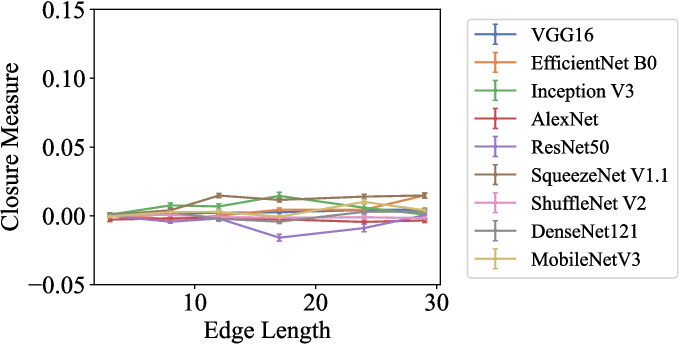
Closure measure plotted against edge length (in pixels) for Experiment 1, “Kanizsa Triangles.” The Closure measure ranges from −1 to 1, with larger values indicating a stronger Closure effect

#### Measurement

The Closure measure is defined as the difference in similarity between a valid Kanizsa triangle and a complete triangle, compared to an invalid Kanizsa triangle and a complete triangle. A large positive value suggests a strong Closure effect, while a value close to 0 indicates no Closure effect. The rationale is that if a model exhibits the Closure effect, it would perceive an illusory contour of a triangle in the valid condition, leading to more similar representations of a valid Kanizsa triangle and a complete triangle. We use the same models and layers as in the Triangle Segment Completion part of this experiment.

#### Results and Discussion

Results (Fig. 7) show a negligible Closure effect across all models and edge lengths. Multivariate linear regression analyses revealed significant but weak effect sizes (adjusted $R^2 <.16$) on the Closure measure for Inception V3, AlexNet, ResNet-50, ShuffleNet V2, DenseNet-121, and MobileNet V3. Among these, the predictive effect of edge length on the Closure measure is significant for ShuffleNet V2, DenseNet121, and MobileNet V3, but their coefficients are much smaller than those obtained in the previous experiment ($b <.0004$, $p <.001$). The regression models for VGG16, EfficientNet B0, and SqueezeNet V1.1 are significant ($p <.001$) and have medium effect sizes (adjusted $R^2 >.30$). Additionally, edge length significantly predicts the Closure measure in these models ($p <.001$), although the coefficients are smaller than those observed for each model in the Triangle Segment Completion experiment ($b =.0006$, .001, and .0009, respectively).

These results suggest that Inception V3, AlexNet, and ResNet-50 cannot perceive the illusory contour in Kanizsa triangles under the current similarity-based measurement. VGG16, EfficientNet B0, SqueezeNet V1.1, ShuffleNet V2, DenseNet-121, and MobileNet V3 exhibit Closure effects, but these effects are much smaller than those observed for incomplete triangles in Triangle Segment Completion.

However, the possibility remains that the current method is not sensitive enough to detect the capability of the models to perceive illusory contours. For the Kanizsa triangles, we cannot rule out a section of overlap given the larger space the “pac-men” occupy. We tried to address this by randomly adjusting $\theta _{global}$ and center positions. Regardless, some overlap persists, potentially diluting the results. Given that the overlapping area for Kanizsa triangles is much smaller than the non-overlapping area, this measure is still valid, though it should be interpreted with caution. Therefore, we conducted further experiments to investigate whether the models could perceive illusory contours or perform line completion under different measurements. Although the sensitivity of the similarity-based measurement may be limited, enhancing the dataset with Kanizsa triangles is an important contribution and makes a stronger case for models exhibiting the Closure effect. Kanizsa triangles include modal and amodal completion and are therefore a highly complex stimulus considering Closure. Our results show that model performance varies especially for these complex stimuli, justifying their use.

**Fig. 8:**
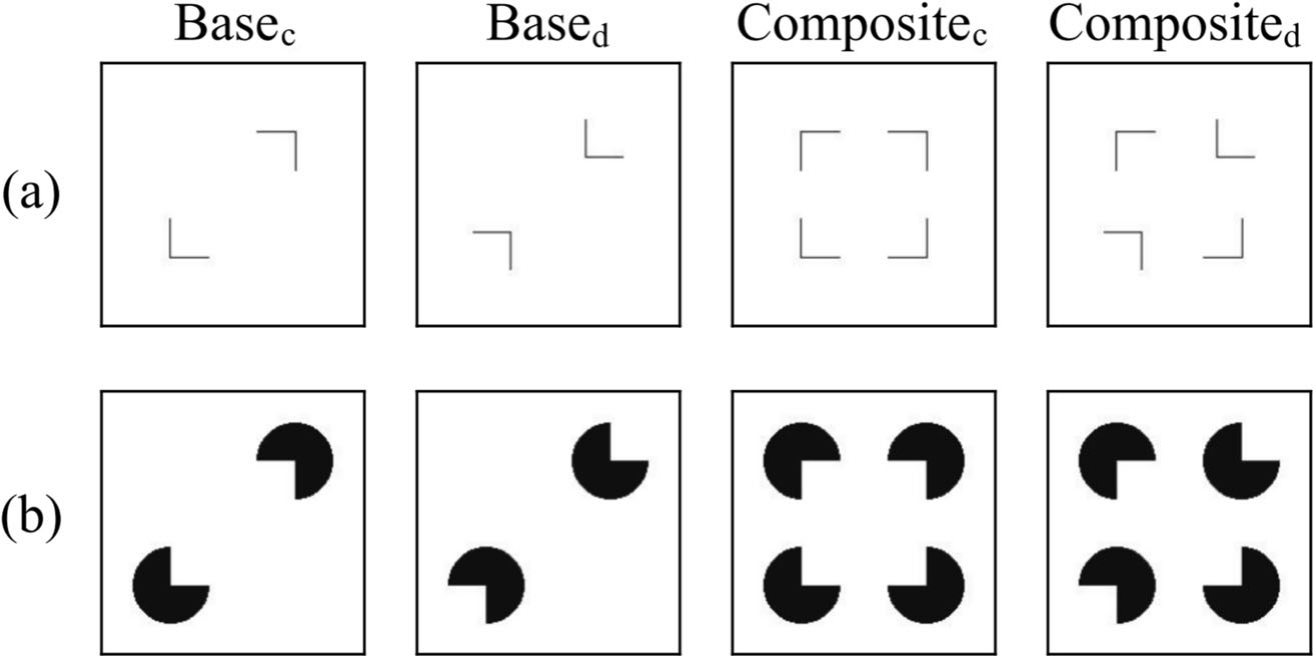
Examples of images used in the second experiment. (**a**) The line segments condition. (**b**) The Kanizsa squares condition. The left two images in each condition are the base pair ($\text {base}_c$ and $\text {base}_d$) and the right two are the composite pair ($\text {composite}_c$ and $\text {composite}_d$)

### Experiment 2: The Configural Effects (CE)-Based Method

Our second experiment uses the concept of configural effects (CE; Pomerantz et al. (1977)), which holds that the perception of a stimulus is influenced by its overall configuration rather than only its individual components. Similarly, Closure enables the perception of the whole figure, even when parts are missing. Closure is a mechanism, which facilitates, in part, the configural effect. In human experiments, participants are asked to find the “odd” image among four presented images, consisting of three identical images and one different image. The pair of different images is either the base pair or the composite pair. If participants perform better on the composite pair than on the base pair (i.e., having shorter reaction times in the discrimination task for the composite pair than for the base pair), they are demonstrating Configural Superiority Effects (CSE) and utilizing Gestalt principles.

Recently revisited by Biscione and Bowers (2023), we have adapted this concept to assess the Closure effect, utilizing a novel dataset in our analysis. Each set in this dataset is composed of two pairs of images: a base and a composite pair. Our hypothesis posits that if a model exhibits Closure, it differentiates composite pairs more easily than base pairs. Consequently, the dissimilarity value of a base pair should be smaller than a composite pair. This would result in a positive difference between the dissimilarity values of the composite pair and the base pair, thus confirming the presence of the Closure effect.

We test this hypothesis on the same CNNs and layers used in the previous experiment. Our approach focuses on analyzing the internal representations of images within these models, rather than directly using the predicted classes. To do this, we compare the activation functions of these CNN layers across different sets of images.

#### Dataset

Our dataset is illustrated in Fig. 8, where we test the Closure effect under two conditions: the line segments condition and the Kanizsa squares condition. For each condition, one set of images consists of two pairs: the base pair and the composite pair. The base pair contains two different images ($\text {base}_c$ and $\text {base}_d$) and either of the two shapes in one image is different from that at the corresponding location in the other image. By appending identical components to each image within a base pair, we construct the composite pair ($\text {composite}_c$ and $\text {composite}_d$).

**Fig. 9:**
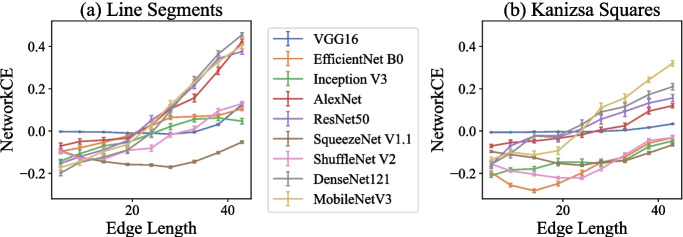
The line graphs with error bars showing network CE values under each edge length condition for all models. The error bars represent the standard errors of each model tested on images across edge lengths in each condition. A positive network CE value indicates the existence of the Closure effect in the corresponding condition, and a larger CE value implies a larger Closure effect. (**a**) The results of the line segments condition and (**b**) the results of the Kanizsa squares condition

Each component in the line segments condition is a fragment of a square, while in the Kanizsa squares condition, each component is an incomplete disk. For each composite image in the line segments condition, the four components are located so that the distance between the centroids of any two neighboring components is the same. The base images contain opposing components in the corresponding composite images. By keeping the distance between centroids instead of vertices to be the same, the distances between the two components in the base pair images would be perceived as the same, and the composite pair images would be perceived as “aligned” by humans. In the line segments condition, the distance between any two neighboring vertices in $\text {composite}_c$ is 95 pixels. In the Kanizsa squares condition, the distance between the centers of neighboring incomplete disks is always 95 pixels. All other parameters are the same across the images in both conditions.

In each condition, the sets of images differ from other sets in their $\theta _{global}$, edge lengths, background colors, and center positions. The $\theta _{global}$ of an image can take any of the following values: 0°, 11.25°, 22.5°, 33.75°, 45°, 56.25°, 67.5°, or 78.75°. Larger degrees are not used because the resulting image would be identical to one generated with a previous $\theta _{global}$. The edge length of an image can be one of the following values: 5, 10, 14, 19, 24, 29, 33, 38, or 43 pixels. In this case, the proportions of the edge length to the side length of the square correspond to 0.9, 0.8, 0.7, 0.6, 0.5, 0.4, 0.3, 0.2, and 0.1, respectively. The background color can be either white or black, and the center position can be either (150, 150) or (134, 134). The last two parameters match those in the first experiment in order to control for irrelevant variables. In total, there are 288 sets of images in each condition, consisting of 288 base pairs and 288 composite pairs. Thus, the number of images in each condition is 1152, and the total number of images used in the experiment is 2304.

#### Measurement

We employ the measurement inspired by the concept of CE (Pomerantz et al., 1977) and implemented by Biscione and Bowers (2023), which is based on the Euclidean distance:

3 $$\begin{aligned} CE=\frac{D^l(composite_c, composite_d) - D^l(base_c, base_d)}{D^l(composite_c, composite_d) + D^l(base_c, base_d)} \end{aligned}$$

where $$ D^l(\textbf{c}, \textbf{d}) = || d^l(\textbf{c}) - d^l(\textbf{d}) || $$.

The key point of our experiment is that if the model exhibits the Closure effect, it should be easier for the model to differentiate the composite pair than the base pair. Consequently, the Euclidean distance of the base pair should be smaller than that of the composite pair. This results in a positive difference between the dissimilarity values of the base pair and the composite pair. Thus, a positive CE value indicates the presence of the Gestalt principle.

#### Results and Discussion

Our results are depicted in Fig. 9. In both conditions, most models show an increase in the CE values, which are positive when the edge length is large enough. Additionally, we conduct a one-sample t-test on the CE values for each model at each edge length in both the line segments and Kanizsa squares condition.

##### The line segments condition

The average CE value for AlexNet is significantly larger than 0 when the edge length is 28 pixels or more ($p <.001$), reaching a mean of 0.042 at the edge length of 43 pixels. In ResNet-50, the average CE is significantly larger than 0 when the edge length is 24 pixels or more ($p =.024 <.05$ at 24 pixels, and $p <.001$ for larger edge lengths), with a maximal average CE of 0.376. DenseNet-121 presents a significant Closure effect ($p <.001$ for edge lengths of 28 pixels or more), with the largest average CE being 0.455. The average CE for MobileNet V3 is significantly positive when the edge length is 28 pixels or more, achieving a maximum value of 0.405. VGG16, EfficientNet B0, Inception V3, and ShuffleNet V2 show significantly positive average CE values when the edge length is sufficiently large, although the values are not as high as those in AlexNet, ResNet-50, DenseNet-121, and MobileNet V3. SqueezeNet V1.1 exhibits significantly negative CE values ($p <.001$), indicating that it does not demonstrate the Closure effect.

The results above indicate that all models tested in the experiment, except for SqueezeNet V1.1, exhibit the Closure effect in the line segments condition, provided that the removed portion of a side in the square is not too large. Among those employing the Closure principle in processing the images, AlexNet, ResNet-50, DenseNet-121, and MobileNet V3 exhibit larger average CE values compared to the other four models, indicating stronger Closure effects. These models may perform better at differentiating aligned and disordered square segments, demonstrating a stronger ability to utilize the Closure principle.

**Table 1:** Summary of results indicating the presence of the Closure effect in each model across two experiments

Model	V1	Similarity-based method		CE-based method	
		Incomplete	Kanizsa	Incomplete	Kanizsa
		triangles	triangles	squares	squares
VGG16	0.538	✔ ($r \le 0.9$)	✓ ($r \le 0.8$)	✔ ($r \le 0.2$)	✔ ($r \le 0.3$)
EfficientNet B0	0.492	✔ ($r \le 0.8$)	✓ ($r \le 0.7$)	✔ ($r \le 0.4$)	✗
Inception V3	0.496	✔ ($r \le 0.9$)	✗	✔ ($r \le 0.3$)	✗
AlexNet	0.508	✓ ($r \le 0.7$)	✗	✔ ($r \le 0.4$)	✔ ($r \le 0.3$)
ResNet-50	0.511	✗	✗	✔ ($r \le 0.5$)	✔ ($r \le 0.4$)
SqueezeNet V1.1	0.158	✔ ($r \le 0.9$)	✓ ($r \le 0.9$)	✗	✗
ShuffleNet V2	0.446	✔ ($r \le 0.9$)	✓ ($r \le 0.4$)	✔ ($r \le 0.2$)	✗
DenseNet-121	0.497	✓ ($r \le 0.7$)	✓ ($r \le 0.6$)	✔ ($r \le 0.4$)	✔ ($r \le 0.4$)
MobileNet V3	0.499	✗	✓ ($0.5 \le p \le 0.6$)	✔ ($r \le 0.4$)	✔ ($r \le 0.4$)

We include brain scores on V1 (primary visual cortex) for general discussion. A checkmark indicates the presence of the Closure effect, with thicker checkmarks denoting a moderate to large effect size and thinner checkmarks indicating a small effect size. Crosses indicate no Closure effect has been found. *r* refers to the removal percentage and $r \le a$ suggests that the Closure effect only exists when the removal percentage is no larger than *a*

When the edge length is small (i.e., shorter than 24 pixels), all models except for VGG16 have average CE values that are significantly lower than 0. These results suggest that for those models, distinguishing between the two composite images becomes more difficult than differentiating the base pair. This difficulty may arise because the added components in the composite pair increase the image processing cost, making it harder for the models to distinguish between the images in the composite pair.

The performance decrease with edge length is something that is also present in psychophysical experiments with human participants investigating Closure. There is evidence to suggest that human closure performance is rather more continuous than strictly binary (Elder & Zucker, 1993). Work on fragmented object outlines has shown that at fragmentation levels of 15%, 20%, 25%, and 30% visibility of the total object outline, correct identification of the object increased as the visible percentage increased (Panis et al., 2008).

##### The Kanizsa squares condition

The average CE value for MobileNet V3 is significantly larger than 0 when the edge length is no less than 28 pixels ($p <.001$1), with the highest average CE being 0.321. DenseNet-121 also shows significantly positive average CE values at edge lengths of 28 pixels or more ($p <.001$), with a maximum average CE of 0.210. ResNet-50, AlexNet, and VGG16 show significantly positive average CE values when the edge length is larger or equal to 28, 33, and 33 pixels ($p <.001$), respectively. The largest average CE values for the three models are 0.156, 0.120, and 0.034, respectively.

These results indicate that for MobileNet V3, DenseNet-121, ResNet-50, AlexNet, and VGG16, the valid and invalid Kanizsa squares in the composite pair are represented as more different than the images in the base pair. As discussed above, a model employing the Gestalt principle might have more differentiated representations of an image that induces the Gestalt effect and an image that does not, compared to the representations of a base pair (Biscione & Bowers, 2023). Thus, these models exhibit the Closure effect when the edge length is large enough.

Other models (i.e., EfficientNet B0, Inception V3, Squeeze-Net V1.1, and ShuffleNet V2) show significantly negative average CE values no matter how large the edge length grows. Models that exhibit the Closure effect also have negative CE values when the edge length is small. Thus , in these conditions, the models cannot facilitate the process of the Kanizsa square in the composite pair; rather, their processing of images might be impaired by the added information in the composite pair compared to the base pair.

## General Discussion

We present an overview of our results from both experiments in Table 1. Notably, VGG16 and DenseNet-121 consistently demonstrate the ability to utilize the Closure principle across both experiments. EfficientNet B0, SqueezeNet V1.1, and ShuffleNet V2 exhibit the Closure effect under the similarity-based method but do not show consistent results with the CE-based measurement. Conversely, AlexNet, ResNet-50, and MobileNet V3 show robust Closure effects under CE-based measurements but not with the similarity-based method. Inception V3, however, displays the Closure effect only in the line segments conditions and not in the Kanizsa shapes conditions.

We observe that changing the dataset from line segments to Kanizsa shapes for the same measurement results in a decreased proportion of models exhibiting the Closure effect, accompanied by smaller effect sizes (i.e., the Closure measure does not increase significantly in the figure, and regression analysis gives a small coefficient for edge length). This finding is interesting in front of the ongoing discussion in vision research on modal and amodal completion being a unified mechanism (Shipley & Kellman, 1992; Kellman et al., 1998), or two different perceptual mechanisms (Spehar & Halim, 2016). The added difficulty for Neural Networks to classify shapes using modal completion could support the hypothesis of distinct mechanisms. At the very least, it is evidence for different mechanisms within convolution neural networks. Specifically, in the similarity-based method, the Closure effect is detected in 7 out of 9 models with line segments, with only 2 models displaying relatively small effect sizes. In contrast, all 6 models showing the Closure effect with Kanizsa triangles exhibit small effect sizes. With the CE-based method, 8 out of 9 models show the Closure effect for line segment images, but this number drops to 5 for Kanizsa squares.

Our findings show that CNNs may possess stronger capabilities for amodal completion and weaker abilities for modal completion. This could explain why some models exhibit the Closure effect in the line segments condition but not in the Kanizsa shape condition, which requires both types of completion, and why the effect is stronger with line segments. Additionally, CNNs have only been trained to assign a single class to an image rather than categorizing it into multiple classes. This means that CNNs consider all image features when determining the class, including those of the black disks in Kanizsa images, which are irrelevant to the perception of illusory contours and may introduce noise.

Notably, when comparing neural networks to human perception, it is natural to observe both similarities and differences. While Closure is considered a mid-level visual phenomenon in human vision, this does not imply that it must emerge at a similar stage in artificial networks. The internal mechanisms of the brain and neural networks differ fundamentally, but both aim to accomplish similar tasks—object recognition being one of them. Our goal is not to equate their internal workings, but to evaluate whether their outputs can exhibit comparable perceptual behavior.

We also examined the Brain Score (Schrimpf et al., 2018), a widely used metric for measuring the alignment of neural networks with the human brain.^2^ This score quantifies how closely model predictions correspond to biological data, such as neural activity or behavior, as evaluated by the specific metrics of each benchmark. The scores are normalized values ranging from 0 to 1, with 1 indicating the closest alignment. We found no clear relationship between the similarity-based method results and the Brain Scores. However, scores on the “neural” aspect, especially on “V1” (primary visual cortex), are strongly related to the CE-based method results. Models with low “V1” scores are less likely to exhibit the Closure effect with the CE-based method. This suggests that models more aligned with V1 are more likely to show the Closure effect, supporting the idea that early stages of visual processing involve configural information (Fox et al., 2017; Moors et al., 2020).

## Conclusion

We conducted a detailed analysis of the Closure effect in CNNs and aimed to settle the ongoing debate in the literature. Previous studies have lacked consensus, with some reporting evidence of Closure while others opposing it, often relying on a limited number of CNNs and experiments. While their feedforward-only architecture might not seem an intuitive choice for investigating mechanisms derived from the human brain, we argue that an even stronger case could exist for studying the architecturally simpler CNNs. If a “simple” CNN relies on mechanisms similar to perceptual grouping in humans, without recurrent connections, this represents a significant finding for the fields of AI, Neuroscience, and Psychology, contributing to our understanding of grouping mechanisms. Conversely, if CNNs do not inherently exhibit such behavior, incorporating these mechanisms could enhance their performance and reduce their vulnerability to adversarial attacks. By using stimuli traditionally utilized to assess Closure in humans, our experiments provide a natural bridge for exploring these phenomena in CNNs.

In addition to CNNs, investigating ViTs represents a promising direction for future research. ViTs incorporate attention mechanisms, and there are complex interrelationships between attention mechanisms and perceptual grouping in the human brain (Wu et al., 2023). Therefore, ViTs require separate investigation. Nonetheless, we conducted preliminary experiments on ViTs. Our experiments with the ViT (ViT-Base, Patch Size 16, 224*tyimes*224) reveal results consistent with CNNs under the line segments condition in our similarity-based method. However, in the CE-based method, we observed a different pattern: while CNNs tend to exhibit Closure effects in line segment conditions but not in Kanizsa square conditions, the ViT demonstrates a different behavior. Accordingly, a detailed study of ViTs could provide deeper insights into their perceptual capabilities and their relationship to human vision.

Finally, we evaluated current CNNs without modifications to assess their inherent ability to perform Closure, given their proficiency in image classification. Rather than training CNNs specifically to perform Closure, we investigated whether this capability arises naturally in these models. As Closure is an innate human mechanism—enabling object detection through contour completion and integration—CNNs with object detection capabilities should either inherently possess this mechanism or lack it. Our exploration highlights the significant challenges these models still face. By comparing CNN performance to human vision, we deepen our understanding of their limitations. In future work, training CNNs to perform Closure could help improving their performance and robustness.

## Data Availability

Our code and dataset will be made available on GitHub.

## Footnotes

1 Our code and dataset will be made available on GitHub.

2 https://www.brain-score.org/vision/

## Material Availability

Our code and dataset will be made available on GitHub.

## Code Availability

Our code and dataset will be made available on GitHub.
